# A novel pleural-bladder pump for the management of recurrent malignant pleural effusions: a feasibility animal study

**DOI:** 10.1186/s12931-020-01447-4

**Published:** 2020-07-15

**Authors:** S. Laroumagne, J. Guinde, S. Berdah, H. Dutau, J. Capel, P. Astoul

**Affiliations:** 1 Department of Thoracic Oncology, Pleural Diseases and Interventional Pulmonology – Hôpital Nord, Marseille, France; 2 LBA-UMRT24, Aix-Marseille Université, Marseille, France; 3 Sequana Medical AG, Zurich, Switzerland; 4 Aix-Marseille University, Marseille, France

**Keywords:** Malignant pleural effusion, Pleural pump, Thoracoscopy, Pleural drainage

## Abstract

**Background:**

Recurrent malignant pleural effusions (MPE) are common and associated with significant morbidity in cancer patients. A new pump connecting the pleural cavity and the bladder may have application for the management of recurrent MPE. In a pre-clinical study, we investigated the utility of this pump in healthy pigs.

**Methods:**

A novel pump system (Pleurapump® system) was inserted into four pigs under general anaesthesia. A tunnelled-pleural catheter was connected to a subcutaneously implanted pump while the urinary bladder was connected by percutaneous technique. Animals were ventilated mechanically and pump functioning was tested using a range of ventilation parameters and spontaneous breathing. Fluid was added to the pleural space to mimic pleural effusion and to assess the effectiveness of the pump at removing fluid to the bladder.

**Results:**

The ‘pleurapump’ system successfully transported fluid from the pleural cavity to the bladder. Pressure variations caused by respiration and variations in the amount of fluid in the pleural cavity had no impact on the pumping. Pumping stopped when the pleural cavity was drained.

**Conclusion:**

This pump can be implanted into pigs and successfully removed fluid from the pleural cavity to the bladder and may represent a new treatment for management of recurrent MPE. Evaluation in humans is planned.

## Introduction

Malignant pleural effusion (MPE) is a frequent feature of disseminated or advanced cancer [1]. MPE most commonly originates from a primary tumour of the lung (~ 40%), although breast cancer, lymphomas, genitourinary tract tumours and gastrointestinal tract tumours are also common sites of origin [2]. Among various potential mechanisms leading to the development of MPE studies have also suggested that interactions between pleural-based tumour cells, host vasculature and immune system may result in increased fluid production via enhanced plasma extravasation into the pleural space leading to MPE [3, 4].

The consequences of MPE, such as dyspnoea and cough, substantially decrease patients’ quality of life and as such, alleviating these symptoms is one of the major goals of therapy. Commonly used therapeutic options include the removal of the pleural fluid or pleurodesis using talc. British Thoracic Society guidelines recommend indwelling pleural catheter insertion for selected patients and pleural symphysis using graded talc as treatment options for the management of recurrent MPEs [5]. Both methods have been shown to improve symptom control, relieve patient-reported dyspnoea and improve patient’s quality-of-life [6, 7] and research suggests that there are no significant differences between techniques regarding symptom control, subjective relief from MPE-related dyspnoea, quality-of-life and health-care costs [8]. Despite the benefits of these interventions, both procedures are not without limitations; talc pleurodesis requires hospitalization while indwelling pleural catheterization requires ongoing drainage at home which can be unattractive, intrusive and uncomfortable and sometimes painful, especially in patients with trapped lung. As such, alternatives for the management of recurrent MPE are needed. A new pump system may have application for the management of recurrent malignant pleural effusions and represents a modification to the existing European Regulatory Approved alfapump® (automated low flow ascites pump) system used to automatically move the ascite from the peritoneal cavity to the urinary bladder where it is eliminated through normal urination [9]. The current study aimed to demonstrate that a modified alfapump system (Pleurapump® system) could be successfully implanted into animals presenting an experimental pleurisy and automatically remove pleural fluid from the pleura to the bladder before urination under different controlled physiological parameters mimicking the different stages of pleural effusion.

## Material and methods

### *Pleurapump*® system

**Fig. 1:**
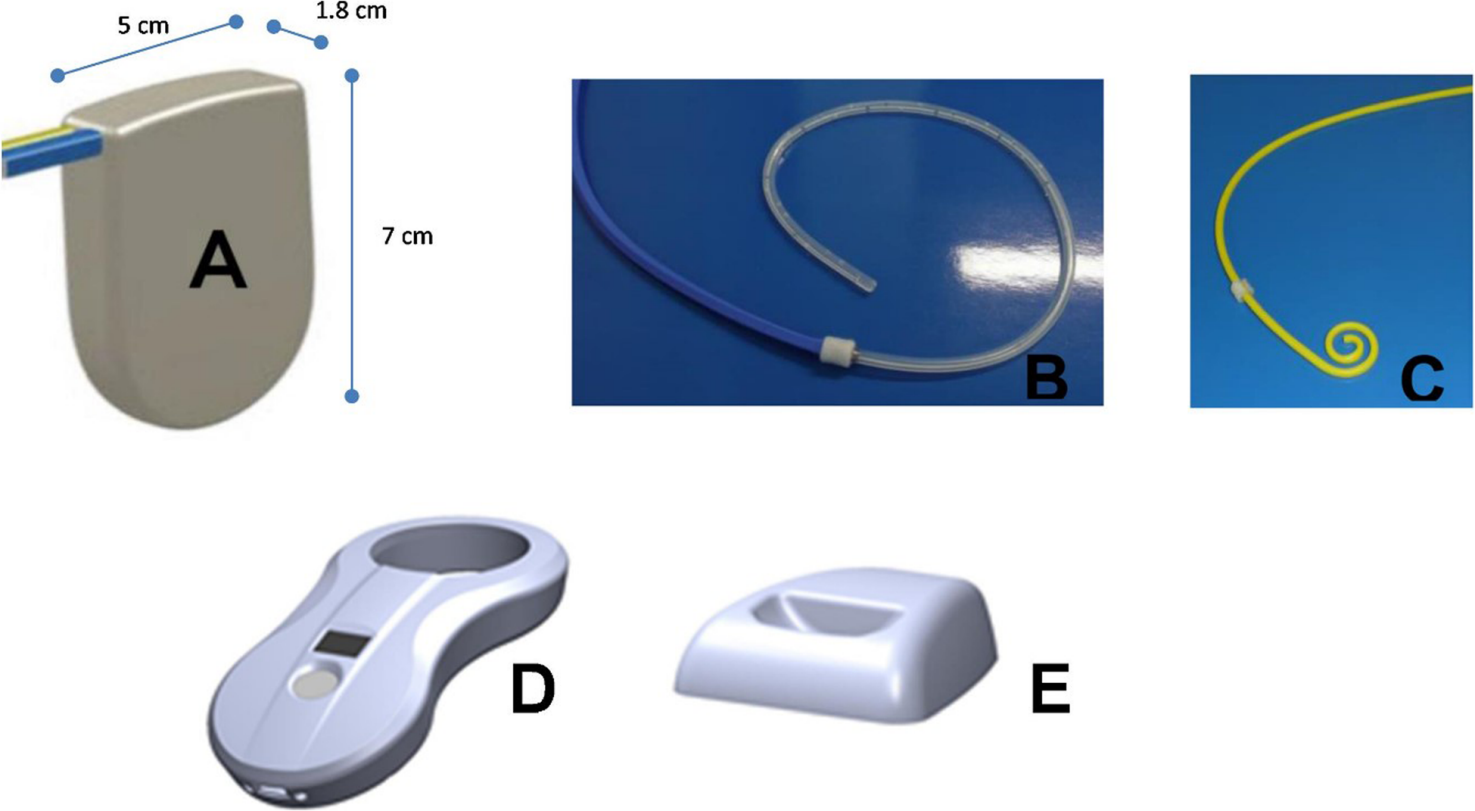
The alfapump system components - alfapump (**a**), pleural catheter (**b**), bladder catheter (**c**), smart charger (**d**) and docking station (**e**)

**Fig. 2:**
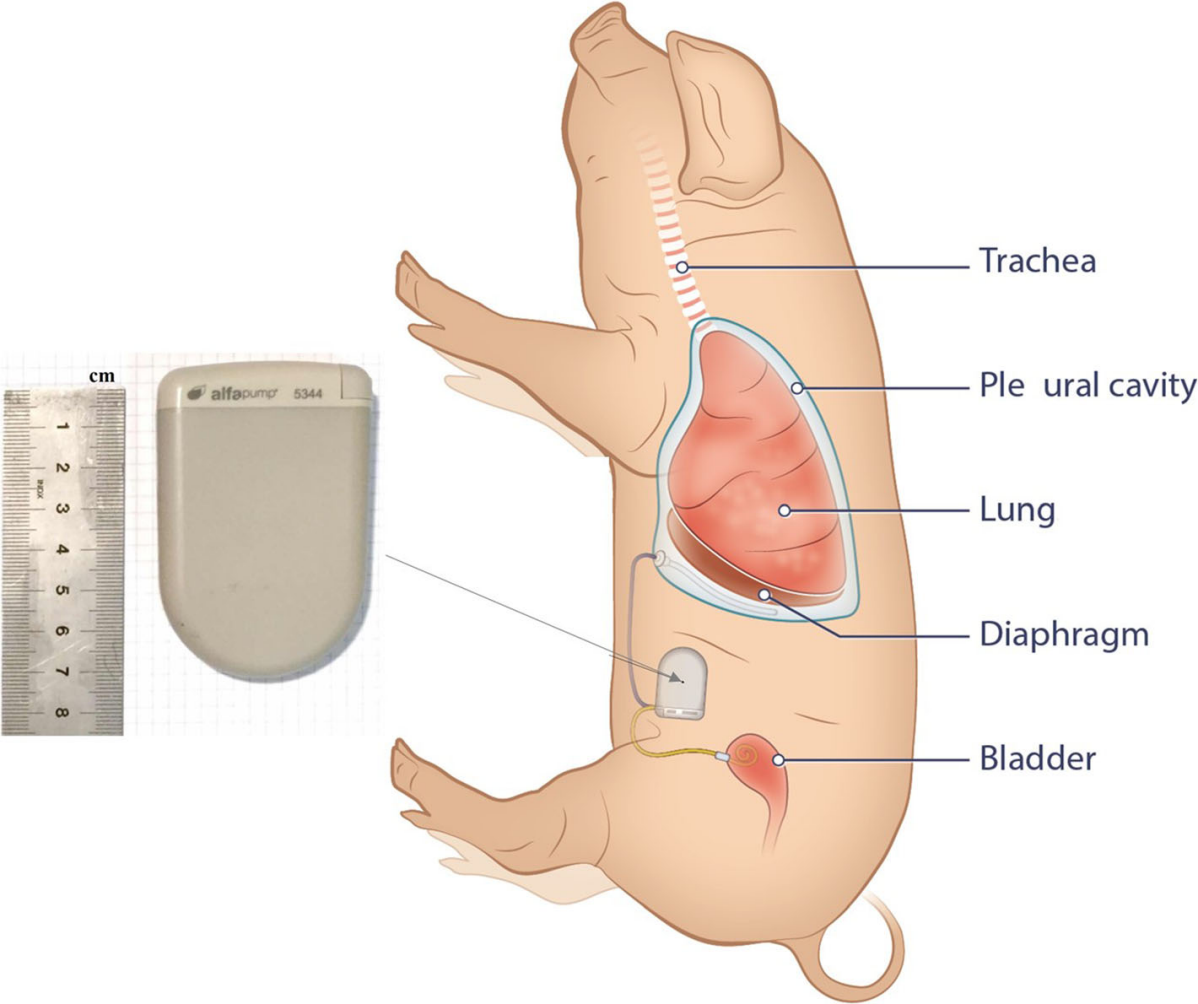
Pig Alfapump placement. The blue catheter connects the pleural cavity to the alfapump while the yellow catheter connects the alfapump to the bladder

The modified alfapump® system is a subcutaneous device with a rechargeable battery, allowing fluid to be moved from the pleural space to the urinary bladder and then urination. The alfapump only allows unidirectional flow through a one-way valve. This to avoid urine flowing into the pleural cavity. As such, the system is designed to provide a permanent and convenient solution for management of recurrent pleural fluid effusions. The modified *alfapump system* (Figs. 1 and 2) consists of an implantable sealed housing (A) containing an internally powered pump with supportive electronic components and circuits; two implantable silicone catheters with dacron cuffs to anchor them in position – a pleural catheter (15Fr, 80 cm; B) and a bladder catheter (15Fr, 44 cm; C) – and a non-implantable charger (D and E). One of the catheters is located within the pleural space and the other is implanted in the urinary bladder, with the pump and battery implanted subcutaneously into the abdomen. The catheters are tunnelled subcutaneously from their respective insertion location to the pump pocket without component protruding the skin. The device moves the fluid from the pleural space to the bladder through the two catheters, via the pump. Unlike peritoneal cavity pressure, which has a positive pressure that changes very slowly, the pleural cavity is exposed to dynamic negative pressure gradients caused by respiration. The difference in physiological conditions of the pleural and peritoneal space required the creation of new pumping algorithms for use in the alfapump to allow for safe and effective removal of fluid from the pleural cavity. These pumping algorithms control fluid pumping in accordance with the parameters predetermined by the physician and is monitored by pressure and position sensors within the device. The implanted battery may be recharged transcutaneously using an inductive (wireless) charger which is also able to programme the pump wirelessly and collect information on pump performance and battery function.

### Study animals

All animal experiments conformed to the European Convention guidelines for the conduct of research using vertebrate animals and with consent from the Centre d’Enseignement et de Recherche Chirurgical (C.E.R.C.), Marseille Animal Ethics Committee [10]. Pigs were selected for experimentation due to the similar level of reactivity of their pleural cavity to that of humans. Male and female pigs (*n* = 4) weighing 28 kg to 35 kg in weight (median 30 kg) were included. Animals were acclimatized for at least 48 h before the start of experimentation and a clinical examination performed to ensure adequate health before selection. All animals were stabled in individual pens and allowed free access to water and a diet based on their weight. Feeding was suspended the day before surgery.

### Surgical procedures

All implant procedures were performed under general anaesthesia using minimally invasive surgical techniques. All animals were premedicated with an intramuscular injection of azaperone (2 mg/kg) and ketamine (10 mg/kg). Two animals were anaesthetized with intravenous propofol (4 mg/kg) prior to orotracheal intubation while two animals were anesthetized with propofol (0.2 mg/kg/min) and sufentanyl (1 μg/kg/h) and left breathing normally. Intubated animals were ventilated mechanically at a frequency of 15 breaths per minute, tidal volume 10 ml/kg, I.E ratio 1:2, peak airway pressure was kept under 30 mmHg. These parameters were modified during pleuroscopy as outlined below. Prior to pleuroscopy, the tidal volume was reduced to 6 ml/kg. Anaesthesia was maintained with propofol (0.2 mg/kg/min) and sufentanyl (1 μg/kg/h) using electrical syringe pulses. Following all procedures, animals were sacrificed using three grams of potassium chloride injected I.V., until confirmation of heart beat cessation.

Following local anaesthesia with 2% lidocaine, artificial pneumothorax was induced by the introduction of ~ 200 mL of air into the pleural space through a smooth ended pleural needle under pleural pressure control at the level of the 4th or 5th intercostal space on the mid-axillary before a gentle 10-mm incision in the line was made. After blunt dissection of the intercostal space, a 7-mm trocar (Wolf®; Richard Wolf, Knittlingen, Germany) was inserted and the pleural cavity explored using a 6 mm telescope (Wolf®; Richard Wolf, Knittlingen, Germany) and the aspect of the lung surface inspected. Placement of a dedicated grade silicone catheter was inserted in the lower part of the pleural cavity under visual control. Insertion of the bladder catheter was done using a modified Seldinger technique. The alfapump system was subcutaneously implanted on the body at a mid-distance between chest and the bladder site using an incision 3 to 4 cm in length at the mid-clavicular line approximately 5 to 6 cm below the costal border. The distance from the pleural catheter insertion point to the pump pocket was at least 10 cm. Catheters were tunnelled subcutaneously from the distal incisions to the pump. The length of the catheters was cut to allow sufficient slack to slide the alfapump into the pocket and for normal movement of the upper body but with care to avoid excessive length as this can lead to kinking of the catheter. To avoid movement of the alfapump and potential kinking of the catheters, the pump was secured in place with two sutures.

### Experimental procedures

The alfapump was tested at two different flow rates (motor speed): 1500 rpm and 5000 rpm for 10 s at each experiment. Of note, an initial rate of 1000 rpm was insufficient to allow the target volume to be transported in the target time. The intra-pleural, ambient and bladder pressures were recorded (built-in pressure sensors) under different controlled physiological conditions as follows:

- In the first two animals, the frequency and amplitude of intermittent positive-pressure ventilation (IPPV) were varied to mimic normal, rapid and slow breathing
  - Normal respiratory rate (15 breath/min) with a tidal volume of 270 ml/breath
  - Respiration with high respiratory rate (28 breath/min) and tidal volume of 140 ml/breath
  - Respiration with low respiratory rate (10 breath/min) and tidal volume of 400 ml/breath
- In the second two animals, experiments were performed under spontaneous breathing with close monitoring

For all three conditions described above, pressure data were recorded with an empty pleural cavity, a half filled (250 ml) pleural cavity and filled (500 ml) pleural cavity; pleural effusion was created by the addition of saline solution to the pleural cavity in a controlled manner. The incidence of negative pressure in the pump inlet, indicative of insufficient fluid volume to complete a programmed fluid transport cycle was recorder as well as successful or unsuccessful pump fluid transport cycle and the volume of fluid transported.

Heart rate of the animals was recorded and X-rays were taken during inspiration and expiration of the lungs to assess potential catheter displacement. Catheters were analysed following experimentation for signs of biological matter. Lungs were inspected visually for signs of possible oedema.

### Statistical analysis

This study was conducted to characterise the pumping parameters of the alfapump under various experimental conditions; no formal statistical testing was planned.

## Results

**Fig. 3:**
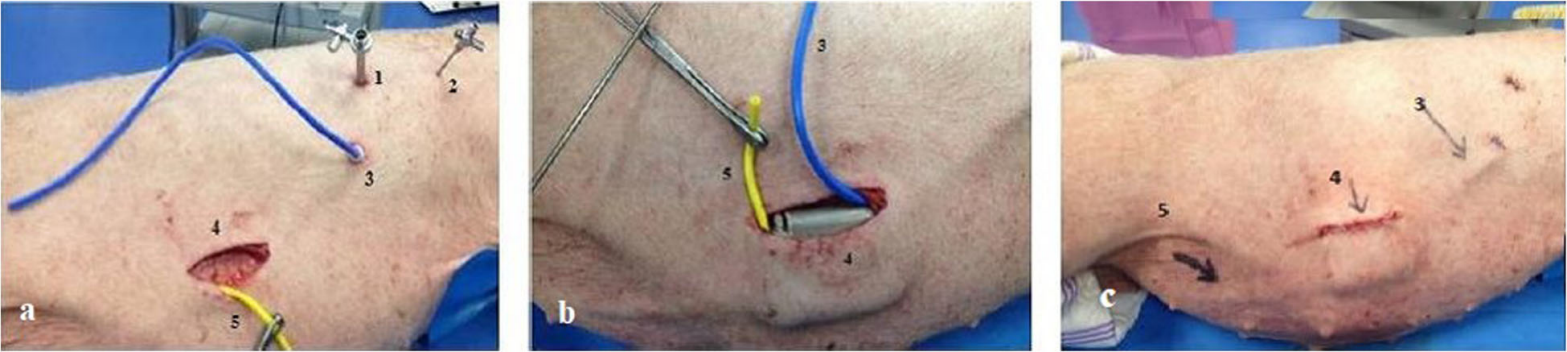
Surgical implantation of the alfapump (1. 7-mm pleural trocar; 2. 2-mm pleural needle; 3. Pleural catheter; 4. Pump Pocket; 5. Bladder catheter). **a** Pleural access (1, 2, 3) and subcutaneous pocket (4) with yellow bladder catheter. **b** Alfapump placement (pleural catheter in blue). **c** Alfapump in place after the end of the procedure

Surgical pump implantation was successfully conducted in four animals. The first procedure took over 1 h with subsequent procedures being complete in less than 40 min. During the first surgery, mal-alignment of the catheters and pump resulted in a kinking of the bladder catheter. This was rectified by tunnelling the catheter in a larger curve such that the bladder catheter entered the pump pocket at the top instead of at the bottom of the pocket. The adaptation lengthened the time of the first procedure. Figure 3 shows the pump positioning before and after pump placement in Animal 1.

### Pump functionality

A summary of the pump functionality in each animal is given in Table 1. Successful fluid transport cycles were completed in 3 animals. In one animal (Animal 2) successful fluid transport cycles were not possible due to technical problems priming the pump prior to implantation and subsequent trapping of air within the pump. Reasons for unsuccessful pump cycles included insufficient fluid present in the pleural cavity, insufficient transport time and excessive bladder pressure. Insufficient transport time resulted from a low flow rate set for the motor of the pump. The low rpm of the pump motor extended the time needed to transport the set target volume and impeded the volume to be transported within the 10 s of activation required by the protocol. Insufficient fluid in the cavity occurred at a higher number of times in Animal 3 than in the other animals in part due to the pleural catheter becoming occluded due to the positioning of the catheter; cleaning was required.

**Table 1:** Pump functionality findings

Animal	Ventilation	Number of pump cycles		Total volume transported (ml)	Number of pump cycle errors		
		Attempted	Successful		Insufficient transport time	Insufficient fluid in cavity	Bladder pressure too high
1	Ventilated	24	33	703.4	6	3	0
2	Ventilated	0	34	0	0	0	0
3	Spontaneous breathing	58	88	1502.8	0	18	12
4	Spontaneous breathing	59	71	1507.5	2	10	0

### Pump presume measurements

**Fig. 4:**
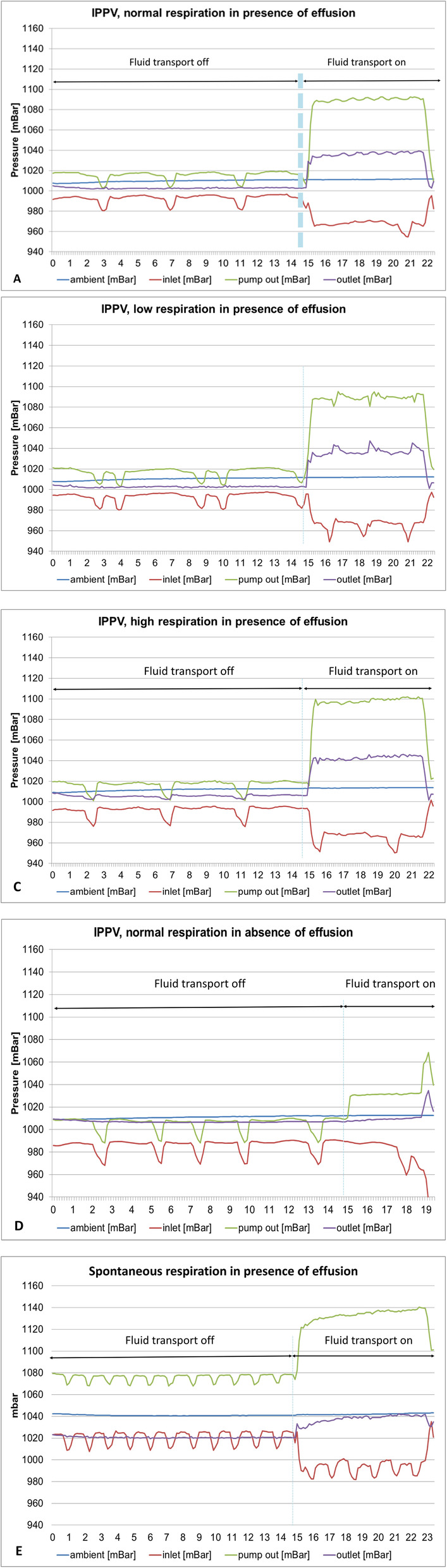
Pressure-time curves during pump activation at different respiratory rates in the absence and presence of fluid in the pleural cavity - Blue line: ambient pressure (mBar), Red line: inlet pressure (mBar), Green line: pump pressure (mBar), Purple line: outlet pressure (mBar). **a**-IPVV, normal respiration in presence of effusion; **b**-IPVV, low respiration in presence of effusion; **c**-IPVV, high respiration in presence of effusion; **d**-IPVV, normal respiration in absence of effusion; **e**-Spontaneous respiration in presence of effusion. IPPV: Intermittent positive-pressure ventilation

**Fig. 5:**
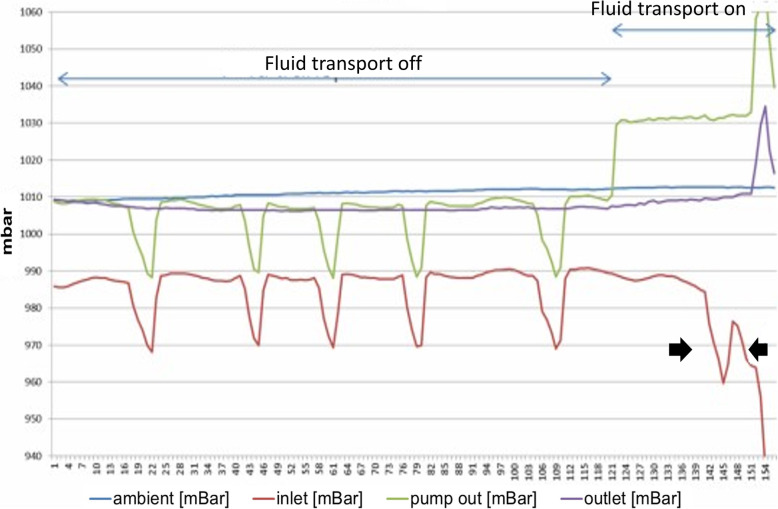
Pressure-time curves during pump activation at high respiration in the absence of fluid illustrating a drop-down resulting in cessation of fluid transport

Selected pressure-time curves during pump activation at different respiratory rates in the absence and presence of fluid in the pleural cavity indicate that successful and comparable pump functioning and fluid transport was achieved at the different respiratory conditions tested (Fig. 4). With regards to the pressure curves, the pump measures and reports in mbar and the reference is the ambient pressure sensor in the pump. Figure 5 shows the pressure-time curves in which a drop-down occurred resulting in cessation of fluid transport. This is illustrated by the fall in pump inlet pressure.

## Discussion

Malignant pleural effusions are associated with significant morbidity and although effective treatments options are available, limitations associated with these procedures mean that alternative treatments options are required. Our findings have shown that the alfapump can be successfully implanted into pigs using standard surgical techniques. The pleural catheter was inserted under visual control during a thoracoscopy as previously described [11]. As such we believe the procedure can be conducted by chest physicians with experience in the placement of pleural drains with assistance from urologists familiar with the placement of supra-pubic catheters. It is vital for the catheters functionality to be aligned in the same plane toward the pump. As both catheters come from opposite directions (thoracic and bladder), they need to be tunnelled under the skin to both arrive from above the pump pocket. This can be achieved by ensuring that the length of the bladder catheter is sufficient to preventing kinking of the catheters when connected them to the pump outlet. Pleural catheter placement will also benefit from the removal of the Dacron. The current study demonstrated that the alfapump system was able to function effectively and safely when implanted into pigs and that the pump successfully removed fluid from the pleural cavity after modifying the pump algorithms for taking into account the dynamic pressure in the pleural cavity. Indeed, motorspeed was set to 1500 RPM (5000 RPM standard), session volume at a maximum of 6 ml (normal range between 6 and 12 ml), and limits for drop down set at 80 mbar (with a standard 200 mbar adaptive drop down switched off). The pump functioned effectively under different controlled conditions simulating different stages of pleural effusion and importantly stopped pumping in the absence of fluid in the pleural cavity Such clinical situation can happen in humans with pleural effusion during cough leading to high pressure and potential compression of catheter along its thoracic and abdominal path. Therefore, due to the relative small volumes being pumped or the catheter crushing, the pleural catheter might not continuously be able to drain fluid. Once fluid accumulates again around the catheter and after the environmental pressure decreased, the fluid can be transported Although pigs were selected based on the similarity of their thoracic cavity to humans, further study in humans are warranted to further validate the surgical procedures used and confirm the utility of this pump in humans.

## Conclusion

A device allowing fluid to be moved from the pleural space to the urinary bladder and then urination can overcome the drawbacks led by the placement of an indwelling pleural catheter and/or a pleural symphysis using graded talc. This study demonstrates that such pump device can be implanted into animals and successfully removed fluid from the pleural cavity to the bladder and may represent a new treatment for management of recurrent malignant pleural effusions. Evaluation in humans is planned.

## Data Availability

The datasets during and/or analysed during the current study available from the corresponding author on reasonable request.

## Abbreviations

MPE: Malignant pleural effusion
Fr: French
C.E.R.C.: Centre d’enseignement et de recherche chirurgical (center for teaching and surgical research)
IPPV: Intermittent positive-pressure ventilation
Rpm or RPM: Revolutions per minute
